# Eribulin alone or in combination with the PLK1 inhibitor BI 6727 triggers intrinsic apoptosis in Ewing sarcoma cell lines

**DOI:** 10.18632/oncotarget.17190

**Published:** 2017-04-18

**Authors:** Lilly Magdalena WeiΔ, Manuela Hugle, Simone Fulda

**Affiliations:** ^1^ Institute for Experimental Cancer Research in Pediatrics, Goethe-University, Frankfurt, Germany; ^2^ German Cancer Consortium (DKTK), Heidelberg, Germany; ^3^ German Cancer Research Center (DKFZ), Heidelberg, Germany

**Keywords:** apoptosis, eribulin, PLK1, Ewing sarcoma

## Abstract

In this study, we investigated the molecular mechanisms of eribulin-induced cell death and its therapeutic potential in combination with the PLK1 inhibitor BI 6727 in Ewing sarcoma (ES). Here, we show that eribulin triggers cell death in a dose-dependent manner in a panel of ES cell lines. In addition, eribulin at subtoxic, low nanomolar concentrations acts in concert with BI 6727 to induce cell death and to suppress long-term clonogenic survival. Mechanistic studies reveal that eribulin monotherapy at cytotoxic concentrations and co-treatment with eribulin at subtoxic concentrations together with BI 6727 arrest cells in the M phase of the cell cycle prior to the onset of cell death. This mitotic arrest is followed by increased phosphorylation of BCL-2 and BCL-x_L_ as well as downregulation of MCL-1, suggesting inactivation of these antiapoptotic BCL-2 family proteins. Consistently, eribulin monotherapy and eribulin/BI 6727 co-treatment trigger activation of BAX, a key proapoptotic BCL-2 family protein, and increase proteolytic activation of caspase-9 and -3. Importantly, overexpression of BCL-2 or addition of the broad-range caspase inhibitor zVAD.fmk significantly rescue eribulin- as well as eribulin/BI 6727-induced cell death. Together, these findings demonstrate that eribulin induces cell death via the intrinsic pathway of apoptosis in ES cells, both alone at cytotoxic concentrations and in combination with BI 6727 at subtoxic concentrations. Thus, our study highlights the therapeutic potential of eribulin for the treatment of ES alone or in rational combination therapies.

## INTRODUCTION

Ewing sarcoma (ES) is the second most common primary pediatric bone tumor predominantly affecting adolescents and young adults that tends to metastasize at an early stage making a cure quite challenging [1–4]. Despite a combination of surgery, irradiation and aggressive chemotherapy, the overall survival of patients with metastasis or relapse is still poor [3, 4]. This calls for the development of new therapeutic strategies to improve survival rates of ES patients and to reduce side effects.

Current chemotherapy protocols for the treatment of ES include cyclic combinations of DNA-damaging drugs such as ifosphamide, actinomycin D, etoposide and doxorubicin as well as the anti-mitotic agent vincristine (VCR) [3, 4]. Dividing cells with DNA damage or defects in mitosis are recognized by cell cycle checkpoints and subsequently eliminated by programmed cell death pathways [5]. Apoptosis represents one of the best characterized forms of programmed cell death and is mediated via two major signaling pathways, i.e. the extrinsic and the intrinsic pathway [6]. This eventually results in the activation of caspases, known as key effector molecules [6, 7]. A number of pro- and antiapoptotic proteins are responsible for the tight regulation of apoptosis. For example, the interaction and the ratio of pro- (i.e. BAX) and antiapoptotic (i.e. BCL-2, BCL-x_L_ and MCL-1) proteins of the BCL-2 family are involved in the control of mitochondrial outer membrane permeabilization, a process that promotes caspase activation, e.g. by the release of mitochondrial intermembrane space proteins [8, 9].

Various kinases play an important role during cell cycle progression including the serine/threonine kinase polo-like kinase 1 (PLK1) [10]. PLK1 is overexpressed in many malignancies including ES [10–12]. We recently reported that PLK1 inhibitors such as BI 6727 provide a new strategy to chemosensitize ES cells and demonstrated that BI 6727 synergizes with VCR to trigger apoptosis in ES cells [13]. An initial screening revealed that this synergistic interaction of BI 6727 extends also to other microtubule-interfering drugs including eribulin [13].

Eribulin is a novel anti-mitotic drug derived from the marine and natural compound halichondrin B with a unique mechanism of action, which has been shown to exert anti-cancer activities at very low doses [14, 15]. Vinca alkaloids and eribulin both bind to the β-tubulin subunit, presumably without overlapping binding sites [15–19]. Whereas vinca alkaloids bind to the ends of microtubules as well as along the microtubule sides [16, 20, 21], eribulin only binds to the microtubules ends with preference to the plus ends [22]. Another unique characteristic of eribulin is its exclusive inhibition of microtubule growth without marked influence on microtubule shortening [14, 22–24], whereas many other tubulin-binding agents affect both parameters [14, 16, 17, 20, 23]. Also, eribulin has been suggested to have a more favorable tolerability profile than most other microtubule-interfering drugs [15, 16, 24, 25]. Preclinical evaluation by the Pediatric Preclinical Testing Program (PPTP) has recently shown that eribulin exerts higher antineoplastic activity in ES xenografts than VCR [25], a classical microtubule-interfering drug that is part of first-line chemotherapy protocols for ES [3, 4]. Eribulin has already entered clinical evaluation (www.clinicaltrials.gov). In a phase III trial eribulin has recently been documented to significantly improve survival of patients with advanced or metastatic soft-tissue sarcomas [26]. In 2016, eribulin has been approved by the Food and Drug Administration (FDA) for patients with unresectable or metastatic liposarcoma who have received an anthracycline-containing chemotherapy beforehand [27].

In light of the documented antitumor activity of eribulin against adult soft-tissue sarcoma as well as childhood malignancies, in the present study we investigated the therapeutic potential and molecular mechanisms of action of eribulin-induced cell death in ES cells, both as a single agent and in combination with the PLK1 inhibitor BI 6727.

## RESULTS

### Eribulin induces cell death in ES cells and cooperates with the PLK1 inhibitor BI 6727

**Figure 1:**
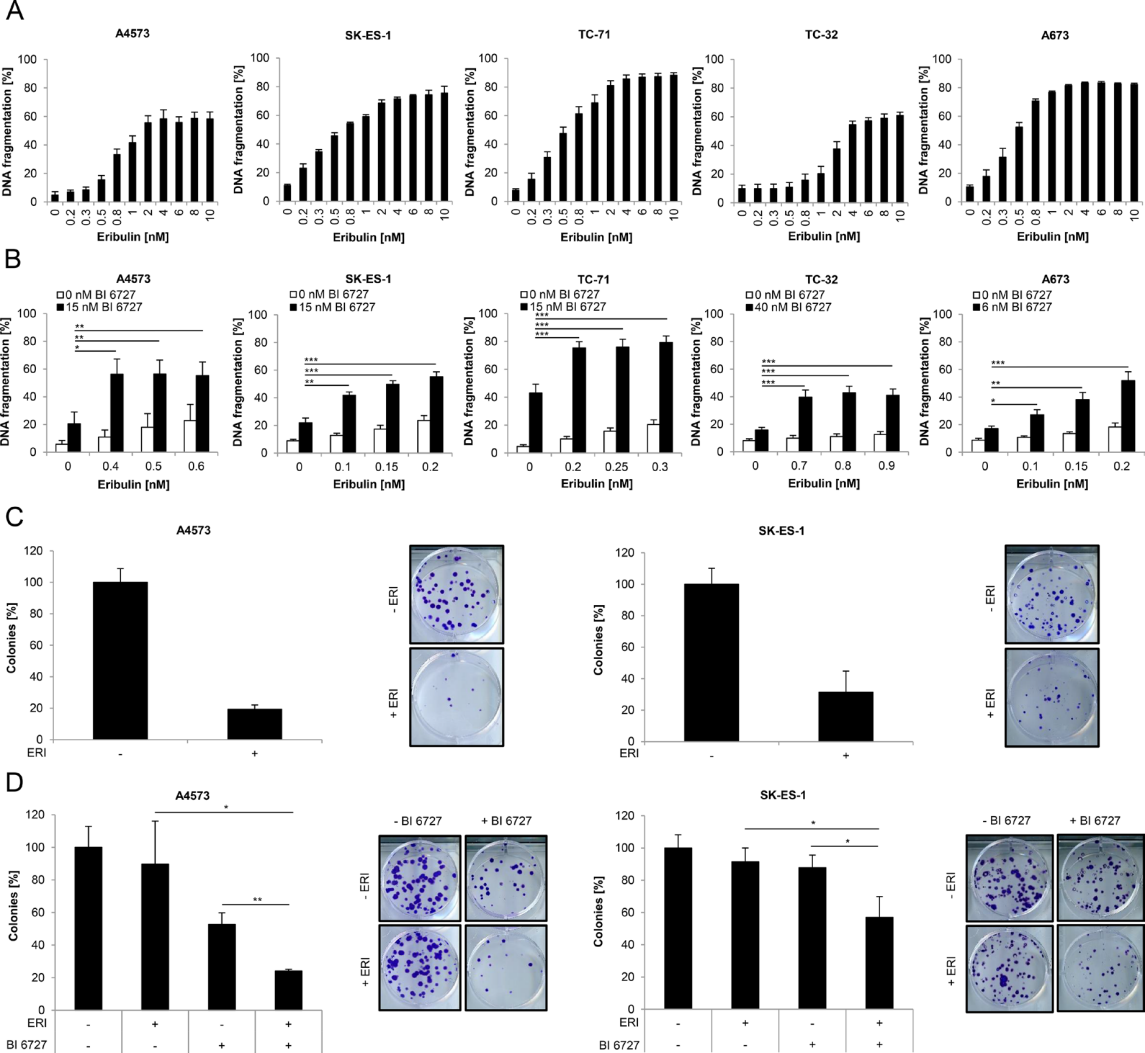
Eribulin induces cell death in ES cells and cooperates with the PLK1 inhibitor BI 6727 (**A**–**B**) ES cells were treated for 48 hours with indicated concentrations of eribulin (A) and/or BI 6727 (B). Apoptosis was determined by quantification of DNA fragmentation of PI-stained nuclei using flow cytometry. Results are expressed as mean with SD of at least three independent experiments performed in triplicate; *p < 0.05; **p < 0.01; ***p < 0.001. (**C**–**D**) A4573 cells were treated for 15 hours with 2 nM eribulin (ERI) (C) or for 18 hours with 0.4 nM eribulin and/or 15 nM BI 6727 (D), SK-ES-1 cells were treated for 15 hours with 1 nM eribulin (C) or for 18 hours with 0.15 nM eribulin and/or 15 nM BI 6727 (D). Subsequently the treatment was replaced by drug-free medium and ES cells were incubated for additional 9 (A4573) or 12 days (SK-ES-1). Colonies were stained with crystal violet solution and counted by using ImageJ software. The colony count is expressed as percentage of untreated controls (left panels) and representative images are shown (right panels). Results are presented as mean with SD of at least three independent experiments performed in triplicate; *p < 0.05; **p < 0.01.

To evaluate the therapeutic potential of eribulin against ES, we assessed DNA fragmentation, a typical parameter of apoptotic cell death [28], upon exposure of a panel of ES cell lines (A4573, SK-ES-1, TC-71, TC-32 and A673) to a broad range of eribulin concentrations. Eribulin dose-dependently triggered an increase in DNA fragmentation in all five ES cell lines already at low nanomolar concentrations (Figure 1A). In addition, subtoxic concentrations of eribulin significantly enhanced BI 6727-triggered DNA fragmentation (Figure 1B).

To address the question as to how cell death induced by eribulin alone at cytotoxic doses and by eribulin at subtoxic concentrations together with the PLK1 inhibitor BI 6727 is mediated on a molecular level we focused our subsequent studies on two ES cell lines, i.e. A4573 and SK-ES-1. To investigate whether eribulin affects long-term survival of ES cells, we performed colony formation assays. Eribulin as single agent markedly suppressed colony formation in comparison to untreated controls (Figure 1C). Moreover, eribulin at low doses significantly cooperated with BI 6727 to reduce colony formation compared to either drug alone (Figure 1D). These results show that eribulin as single agent triggers cell death in a dose-dependent manner and reduces long-term survival of ES cells. Furthermore, subtoxic concentrations of eribulin act together with BI 6727 to induce cell death and to suppress colony formation.

### Caspases partially contribute to eribulin- and eribulin/BI 6727-induced cell death

**Figure 2:**
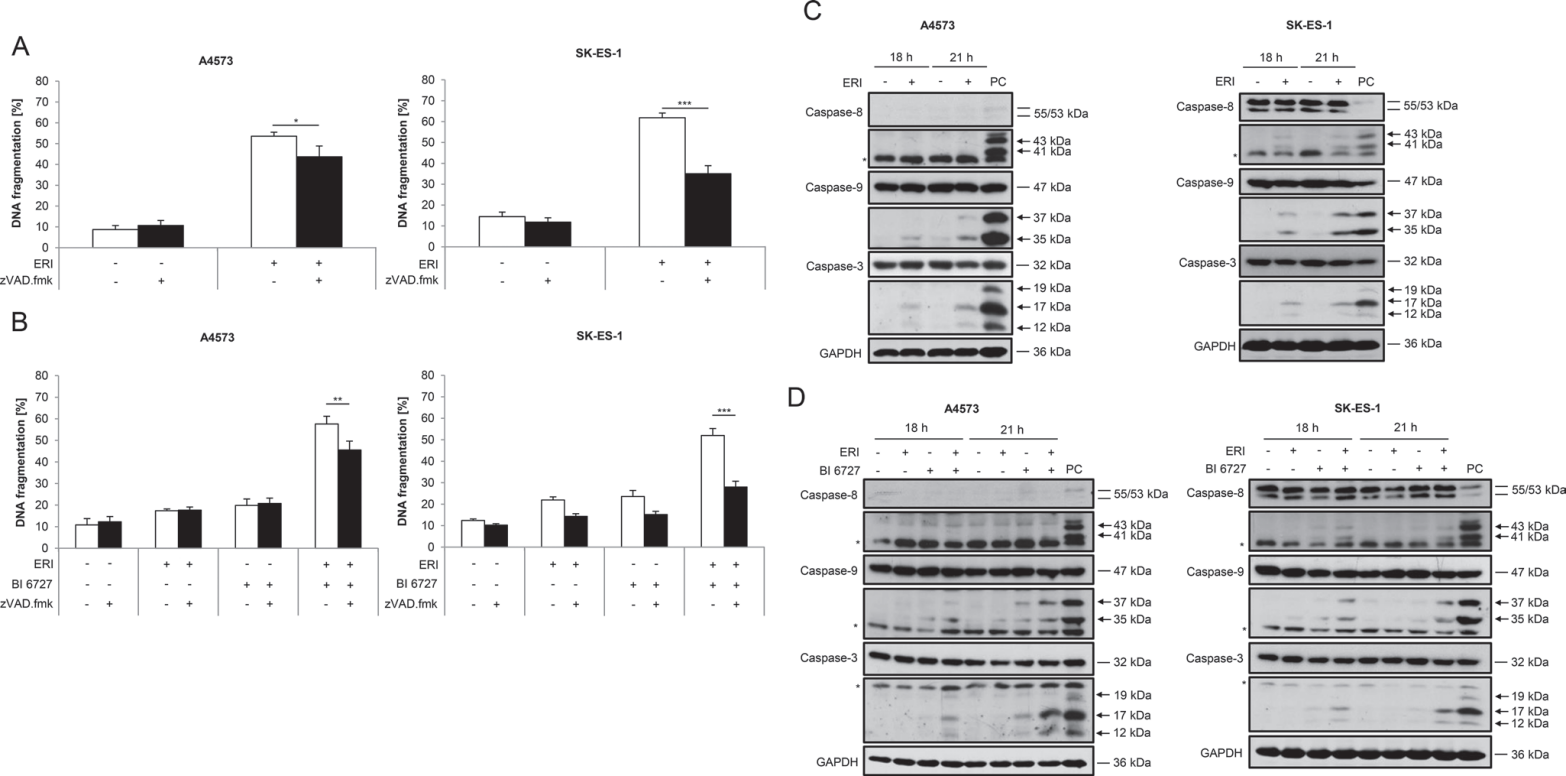
Caspases contribute to eribulin- and eribulin/BI 6727-induced cell death (**A**–**B**) A4573 cells were treated with 2 nM eribulin (ERI) (A) or 0.4 nM eribulin and/or 15 nM BI 6727 (B), SK-ES-1 cells with 1 nM eribulin (A) or 0.15 nM eribulin and/or 15 nM BI 6727 (B) for 48 hours in the presence or absence of the broad-range caspase inhibitor zVAD.fmk (20 μM). Apoptosis was determined by quantification of DNA fragmentation of PI-stained nuclei using flow cytometry. Results are expressed as mean with SD of at least three independent experiments performed in triplicate; *p < 0.05; **p < 0.01; ***p < 0.001. (**C**–**D**) A4573 cells were treated with 2 nM eribulin (ERI) (C) or 0.4 nM eribulin and/or 15 nM BI 6727 (D), SK-ES-1 cells with 1 nM eribulin (C) or 0.15 nM eribulin and/or 15 nM BI 6727 (D) for indicated times. Cleavage of caspase-8, -9 and -3 was examined by Western blotting. A 2 hour treatment of SK-ES-1 cells with 2 μg/mL TRAIL receptor-2 agonistic antibody ETR2 was used as a positive control (PC) for caspase activation for both A4573 and SK-ES-1 cells. Expression of GAPDH was used as loading control. Arrowheads indicate active cleavage fragments, asterisks unspecific bands. Representative blots of two independent experiments are shown.

Since caspases are known as key mediators of apoptosis [6–8], we addressed the question as to whether or not cell death induced by eribulin alone or in combination with BI 6727 is mediated via caspases. To this end, we used the broad-range caspase inhibitor N-benzyloxycarbonyl-Val-Ala-Asp-fluoromethylketone (zVAD.fmk). Of note, the addition of zVAD.fmk significantly rescued DNA fragmentation induced by treatment with eribulin alone (Figure 2A) or in combination with BI 6727 (Figure 2B), although this rescue was partial, especially in A4573 cells. To further explore whether caspases are activated, we monitored cleavage of caspases into active fragments by Western blot analysis. Tumor-Necrosis-Factor-related apoptosis-inducing ligand (TRAIL)-induced caspase activation in SK-ES-1 cells was used as a positive control for both A4573 and SK-ES-1 cells (Figure 2C and 2D). Indeed, treatment with eribulin increased the cleavage of caspase-9 into the active cleavage fragments p37/p35 and of caspase-3 into p17/p12 fragments in both A4573 and SK-ES-1 cells as well as cleavage of caspase-8 into p43/p41 fragments in SK-ES-1 cells (Figure 2C). By comparison, A4573 cells express very low levels of caspase-8, in line with a previous report [29]. Also, eribulin cooperated with BI 6727 to trigger cleavage of caspase-9 and -3 into active cleavage fragments in both cell lines and of caspase-8 in SK-ES-1 cells (Figure 2D). To further confirm the involvement of caspases, we extended our study to a caspase-3/7 activation assay. Notably, eribulin alone at high doses as well as eribulin/BI 6727 co-treatment significantly increased caspase-3/7 activity in both tested ES cell lines compared to their untreated controls or the treatment with either agent alone (Supplementary Figure 1). These experiments show that both eribulin monotherapy and combined treatment with BI 6727 trigger activation of caspases which, at least partially, contributes to the execution of cell death.

### Eribulin and eribulin/BI 6727 co-treatment cause mitotic arrest prior to the onset of cell death

**Figure 3:**
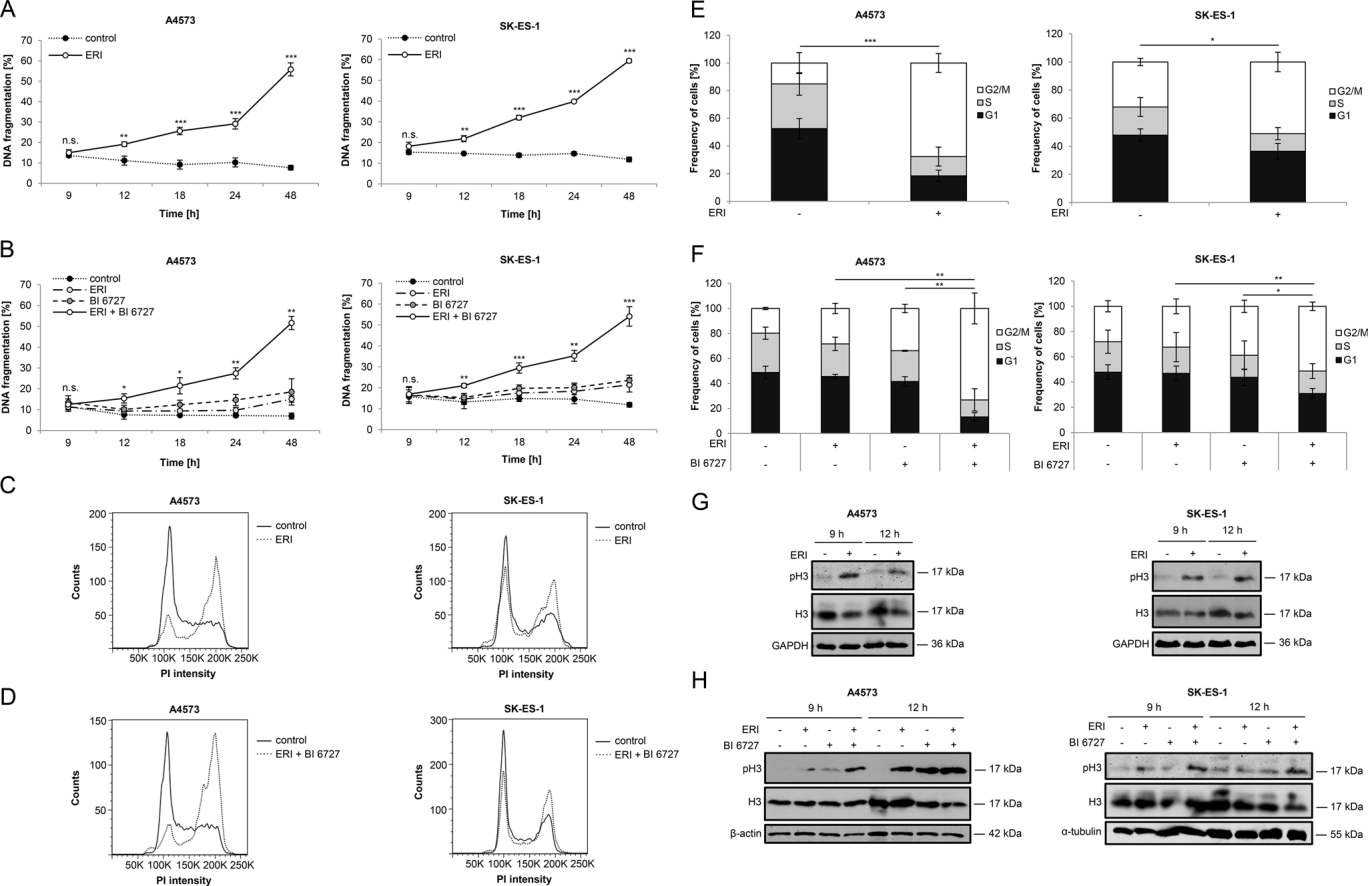
Eribulin and eribulin/BI 6727 co-treatment cause mitotic arrest prior to the onset of cell death (**A**–**B**) A4573 cells were treated with 2 nM eribulin (ERI) (A) or 0.4 nM eribulin and/or 15 nM BI 6727 (B), SK-ES-1 cells with 1 nM eribulin (A) or 0.15 nM eribulin and/or 15 nM BI 6727 (B) for indicated times. Apoptosis was assessed by quantification of DNA fragmentation of PI-stained nuclei using flow cytometry. Results are expressed as mean with SD of at least three independent experiments performed in triplicate; n.s., not significant; *p < 0.05; **p < 0.01; ***p < 0.001 comparing eribulin-treated to non-treated or eribulin/BI 6727-treated to BI 6727-treated cells. (**C**–**F**) A4573 cells were treated with 2 nM eribulin (ERI) (C and E) or 0.4 nM eribulin and/or 15 nM BI 6727 (D and F), SK-ES-1 cells with 1 nM eribulin (C and E) or 0.15 nM eribulin and/or 15 nM BI 6727 (D and F) for 12 hours. Cell cycle analysis was performed by FlowJo software after measurement of PI-stained nuclei using flow cytometry. Results are expressed as representative cell cycle profiles (C and D) or as mean with SD of at least three independent experiments performed in triplicate (E and F); *p < 0.05; **p < 0.01; ***p < 0.001 comparing the frequency of cells arrested in G2/M phase. (**G**–**H**) A4573 cells were treated with 2 nM eribulin (ERI) (G) or 0.4 nM eribulin and/or 15 nM BI 6727 (H), SK-ES-1 cells with 1 nM eribulin (G) or 0.15 nM eribulin and/or 15 nM BI 6727 (H) for indicated times. Expression levels of H3 and pH3 were analyzed by Western blotting. Expression of GAPDH, β-actin or α-tubulin served as loading control. Results are shown as representative blots of two independent experiments.

Since both eribulin as microtubule-interfering drug and BI 6727 as PLK1 inhibitor have been reported to block cell cycle progression [30, 31], we asked whether cell cycle arrest occurs prior or at the onset of cell death. To address this question, we simultaneously monitored the kinetics of cell cycle progression and cell death. This analysis showed that eribulin alone at cytotoxic doses (Figure 3A) or at subtoxic concentrations together with BI 6727 (Figure 3B) induced cell death in a time-dependent manner starting at around 12 hours. Also, we detected a significant increase of cells arrested in G2/M phase already at 12 hours, as represented by tetraploid DNA content, after treatment with eribulin alone (Figure 3C and 3E) or in combination with BI 6727 (Figure 3D and 3F). To distinguish between G2 and M phase, we additionally analyzed phosphorylation levels of histone H3 at serine 10, a specific mitotic marker [32], since analysis of cell cycle distribution by flow cytometry does not allow a discrimination between G2 and M phase. Notably, eribulin alone at high doses markedly increased histone H3 phosphorylation compared to untreated controls at 9 and 12 hours (Figure 3G) prior to the onset of DNA fragmentation (Figure 3A). Also, eribulin and BI 6727 cooperated to cause histone H3 phosphorylation compared to treatment with either drug alone at 9 and 12 hours (Figure 3H). These results show that both eribulin monotherapy at high doses as well as eribulin/BI 6727 co-treatment arrest ES cells in mitosis prior to the onset of DNA fragmentation as a marker of apoptotic cell death.

### Eribulin and eribulin/BI 6727 co-treatment trigger inactivation of antiapoptotic BCL-2 proteins

**Figure 4:**
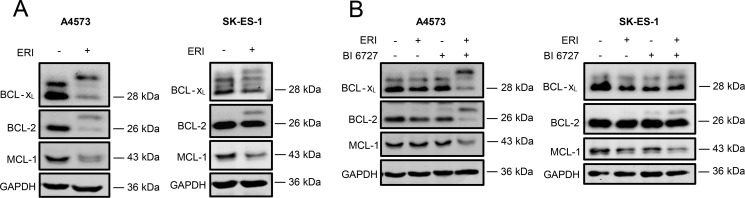
Eribulin and eribulin/BI 6727 co-treatment trigger inactivation of antiapoptotic BCL-2 proteins (**A**–**B**) A4573 cells were treated with 2 nM eribulin (ERI) (A) or 0.4 nM eribulin and/or 15 nM BI 6727 (B), SK-ES-1 cells with 1 nM eribulin (A) or 0.15 nM eribulin and/or 15 nM BI 6727 (B) for 15 hours. Expression levels of BCL-2, BCL-x_L_ and MCL-1 were assessed by Western blotting. GAPDH served as loading control. Results are shown as representative blots of at least two independent experiments.

Since it has been reported that mitotic arrest can lead to post-translational modifications of antiapoptotic BCL-2 family proteins, i.e. BCL-2, BCL-x_L_ and MCL-1 [33–37], we assessed the expression levels and the phosphorylation status of BCL-2, BCL-x_L_ and MCL-1 by Western blot analysis. Interestingly, we detected an upward band shift on SDS-PAGE gel for both BCL-2 and BCL-x_L_ following eribulin single treatment (Figure 4A) and eribulin/BI 6727 co-treatment (Figure 4B), pointing to an increased phosphorylation. To confirm that this upward band shift of BCL-2 and BCL-x_L_ is indeed due to phosphorylation, we incubated lysates from eribulin-treated A4573 cells with or without λ-phosphatase before Western blot analysis. This metal ion-dependent enzyme dephosphorylates phosphorylated amino acid residues (serine, threonine, tyrosine or histidine) of proteins or peptides via hydrolysis [38]. Indeed, addition of λ-phosphatase reversed the eribulin-stimulated upward band shift on SDS-PAGE gel for both BCL-2 and BCL-x_L_ (Supplementary Figure 2A), underlining that these upper bands of BCL-2 and BCL-x_L_ represent their phosphorylated forms. Moreover, treatment with eribulin alone (Figure 4A) and eribulin/BI 6727 co-treatment (Figure 4B) decreased protein levels of MCL-1, although this decrease was slightly weaker in SK-ES-1 cells. To address the question whether or not this downregulation is due to proteasomal degradation as claimed in previous reports upon prolonged mitotic arrest [33, 34], we treated A4573 and SK-ES-1 cells with the proteasome inhibitor bortezomib in addition to eribulin. Of note, addition of bortezomib rescued the eribulin-stimulated downregulation of MCL-1 protein levels (Supplementary Figure 2B), indicating that this decrease in MCL-1 levels is due to proteasomal degradation. Together, these results suggest that eribulin alone at high doses as well as eribulin/BI 6727 co-treatment cause post-translational modifications of key antiapoptotic BCL-2 family members that have been associated with their inactivation.

### BCL-2 overexpression protects from eribulin- and eribulin/BI 6727-induced cell death

**Figure 5:**
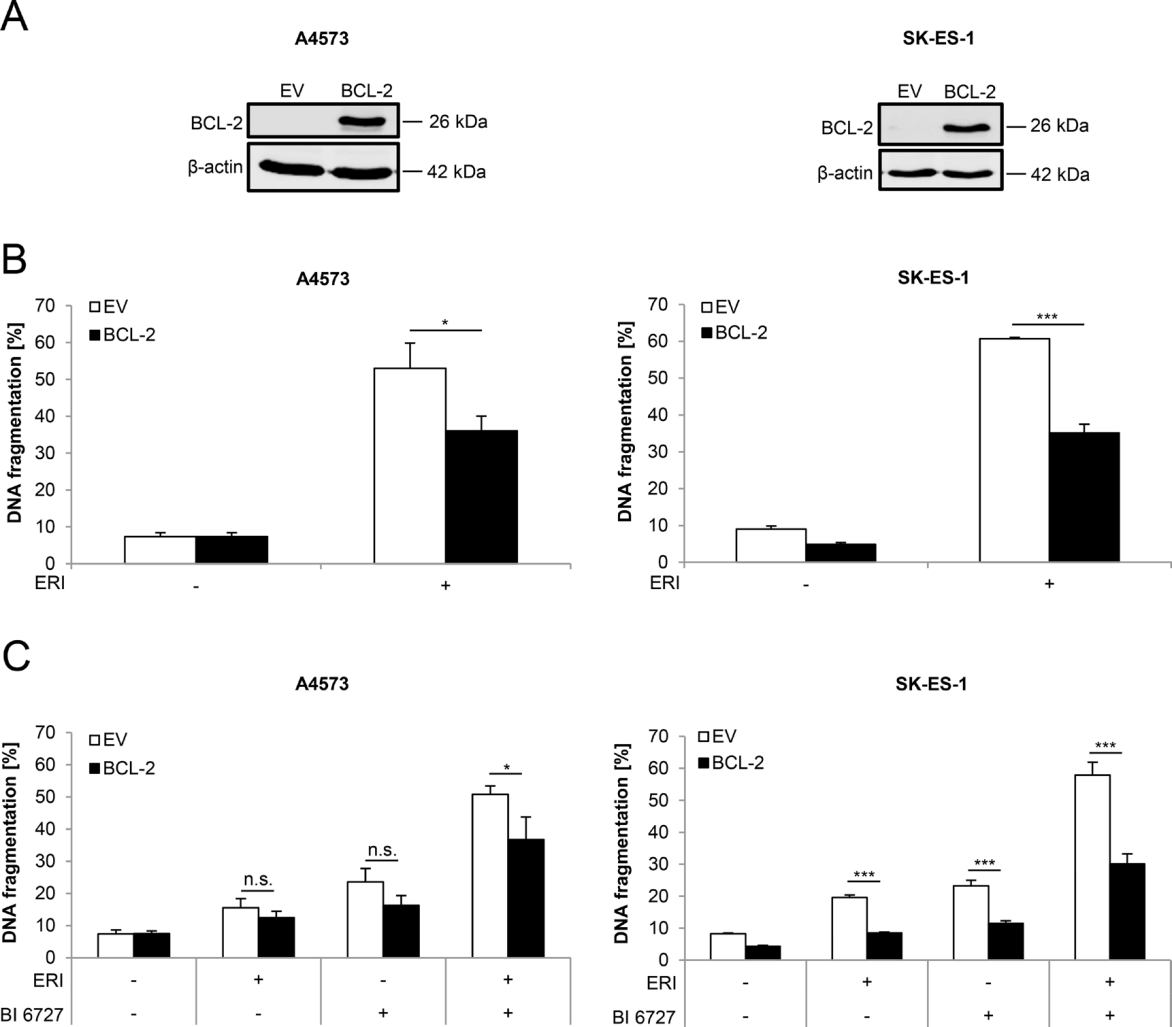
BCL-2 overexpression partially rescues eribulin- as well as eribulin/BI 6727-induced cell death (**A**–**C**) ES cells were stably transduced with empty vector (EV) or murine BCL-2. Transduction efficiency of murine BCL-2 was analyzed by Western blotting. Expression of β-actin served as loading control. Representative blots of two independent experiments are shown (A). Stably transduced A4573 cells were treated with 2 nM eribulin (ERI) (B) or 0.4 nM eribulin and/or 15 nM BI 6727 (C), stably transduced SK-ES-1 cells with 1 nM eribulin (B) or 0.15 nM eribulin and/or 15 nM BI 6727 (C) for 48 hours. Apoptosis was assessed by quantification of DNA fragmentation of PI-stained nuclei using flow cytometry. Results are shown as mean with SD of at least three independent experiments performed in triplicate (B and C); n.s. not significant; *p < 0.05; ***p < 0.001.

To further investigate the role of BCL-2 in regulating eribulin- or eribulin/BI 6727-induced cell death, we generated ES cell lines that stably overexpress murine BCL-2. Ectopic expression of murine BCL-2 was confirmed by Western blot analysis (Figure 5A). Notably, BCL-2 overexpression significantly rescued eribulin- as well as eribulin/BI 6727-induced cell death, although the protection was not complete (Figure 5B and 5C). These findings emphasize the antiapoptotic function of BCL-2 during eribulin- and eribulin/BI 6727-induced cell death.

### Eribulin and eribulin/BI 6727 co-treatment cause BAX activation

**Figure 6:**
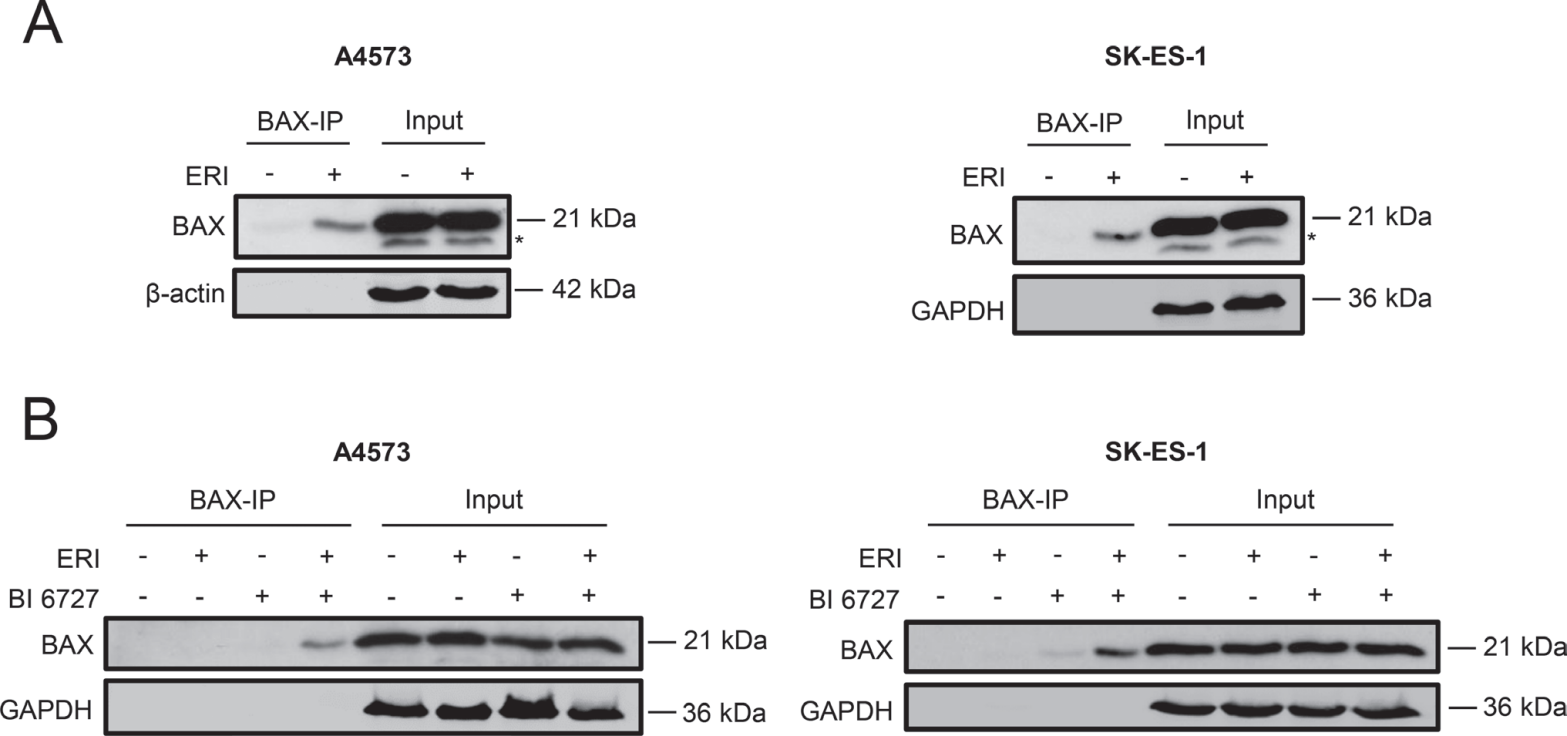
Eribulin and eribulin/BI 6727 co-treatment cause BAX activation (**A**–**B**) A4573 cells were treated with 2 nM eribulin (ERI) (A) or 0.4 nM eribulin and/or 15 nM BI 6727 (B), SK-ES-1 cells with 1 nM eribulin (A) or 0.15 nM eribulin and/or 15 nM BI 6727 (B) for 15 hours. Activation of BAX was assessed by immunoprecipitation of activated BAX using an active conformation-specific antibody (BAX-IP). Expression of β-actin or GAPDH served as loading control. Asterisks indicate unspecific bands. Results are shown as representative blots of at least two independent experiments.

It has been reported that inactivation of antiapoptotic BCL-2 family proteins shifts the balance towards apoptosis resulting in the activation of BAX or BAK, two proapoptotic proteins of the BCL-2 family [8, 9]. Since we detected post-translational changes of antiapoptotic BCL-2 proteins that are associated with their inactivation, we investigated whether treatment with eribulin alone or in combination with BI 6727 stimulates BAX activation. To address this point, we immunoprecipitated activated BAX using an active conformation-specific antibody, as it has been described that BAX undergoes structural changes during activation [8, 9]. Indeed, eribulin at high doses triggered a marked increase of activated BAX levels compared to untreated controls (Figure 6A). Further, eribulin and BI 6727 cooperated to stimulate BAX activation compared to either drug alone (Figure 6B). These results show that both eribulin monotherapy at high doses as well as eribulin/BI 6727 co-treatment stimulate BAX activation.

## DISCUSSION

Eribulin, derived from the marine and natural compound halichondrin B, is considered as a novel and promising microtubule-interfering drug for the treatment of cancer including adult soft-tissue sarcoma and childhood malignancies [25, 26]. In the present study, we therefore investigated the therapeutic potential and the underlying molecular mechanisms of eribulin-induced cell death in ES cells either alone at cytotoxic concentrations or at subtoxic concentrations in combination with the PLK1 inhibitor BI 6727. Here, we demonstrate that eribulin alone dose-dependently triggers cell death in a panel of ES cell lines. Furthermore, we show that subtoxic, low nanomolar concentrations of eribulin significantly enhance BI 6727-induced cell death. In addition to causing increased cell death, treatment with eribulin alone or in combination with BI 6727 also suppresses long-term clonogenic survival of ES cells.

To our knowledge, the elucidation of the underlying molecular mechanisms of cell death induced by eribulin alone or in combination with BI 6727 in ES cells remained so far largely elusive and is therefore of high relevance. Our mechanistic studies reveal the critical role of the intrinsic apoptotic signaling pathway in mediating cell death induced by eribulin alone or in combination with BI 6727. This conclusion is supported by the following pieces of evidence. First, eribulin single as well as eribulin/BI 6727 co-treatment cause inactivation of antiapoptotic BCL-2 family proteins, i.e. phosphorylation of BCL-2 and BCLx_L_ and downregulation of MCL-1. Second, eribulin alone or in combination with BI 6727 stimulates BAX activation. Third, these treatments cause activation of caspase-9 and -3 and DNA fragmentation, typical markers of apoptotic cell death [6, 7, 39, 40]. Fourth, genetic evidence using BCL-2 overexpression emphasizes the importance of BCL-2 inactivation following mitotic arrest and the central role of the mitochondrial apoptosis pathway.

Post-translational modifications of antiapoptotic BCL-2 family members during prolonged mitotic arrest either via phosphorylation (in case of BCL-2 and BCL-x_L_) or via phosphorylation, ubiquitination and subsequent proteasomal degradation (in case of MCL-1) have previously been reported to result in inhibition of their antiapoptotic function [33–37]. Our findings showing that eribulin single as well as eribulin/BI 6727 co-treatment cause mitotic arrest prior to BCL-2 and BCL-x_L_ phosphorylation, MCL-1 downregulation and the onset of apoptosis support the conclusion that these antiapoptotic BCL-2 family proteins become inactivated by phosphorylation during prolonged mitotic arrest. However, the role of BCL-2 phosphorylation has also been controversially discussed [35, 36, 41].

The role of the intrinsic apoptotic signaling pathway is further highlighted by our findings showing activation of proapoptotic BAX as well as caspase-9 and -3 upon eribulin single and eribulin/BI 6727 co-treatment. By comparison, caspase-8 turned out to be dispensable, at least in A4573 cells, which are susceptible to eribulin- and eribulin/BI 6727-induced cell death despite very low levels of caspase-8. Consistently, A4573 cells were shown to be resistant to extrinsic apoptosis induced by the death receptor ligand TRAIL [29, 42]. Caspase-8 has been reported to be epigenetically silenced in some ES cell lines [43]. While our data indicate that caspases contribute to eribulin- and eribulin/BI 6727-induced cell death, caspase-independent mechanisms might also be involved, as the caspase inhibitor zVAD.fmk provides significant, yet not complete protection.

Eribulin has previously been shown to induce cell death associated with apoptotic features in lymphoma and prostate cancer [30], whereas it was reported to trigger non-apoptotic cell death in osteosarcoma cells [44]. This points to tumor type-related differences in eribulin-stimulated signaling pathways that eventually lead to cancer cell death and highlights the relevance of studies to unravel the mechanisms of eribulin-induced cell death in different tumor entities. We previously reported that PLK1 inhibitors and microtubule-interfering agents synergize to trigger apoptosis following prolonged mitotic arrest in ES, rhabdomyosarcoma (RMS) and neuroblastoma (NB) cells [13, 45–47], i.e. tumor entities that have been reported to overexpress PLK1 [11, 12]. In addition to *in vitro* studies, we demonstrated in *in vivo* models of RMS and NB that the PLK1 inhibitor BI 2536 cooperates with VCR or eribulin, respectively, to significantly suppress tumor growth compared to either drug alone [45–47]. Of note, we did not detect additive toxicity of BI 6727 and VCR in a human xenograft mouse model of RMS [45], pointing to some tumor selectivity.

The current study has several important clinical implications. First, our findings indicate that eribulin represents a promising new anticancer drug for the treatment of ES. This notion is emphasized by a previous study of the PPTP demonstrating eribulin's strong anticancer activity in a panel of pediatric xenografts including ES [25]. Additionally, a recent phase III clinical trial showed that eribulin improves the overall survival of patients with advanced or metastatic soft-tissue sarcomas compared to dacarbazine treatment [26]. There is an urgent medical need for novel and more efficient therapeutic strategies for ES as this tumor represents an aggressive malignancy with poor overall survival for patients suffering from relapse or metastatic disease [3, 4].

Second, eribulin might constitute an alternative to VCR because of its more favorable profile, in particular its reduced risk to cause peripheral neurotoxicity [16, 23, 24, 48]. Microtubule-interfering drugs are an important pillar in cancer therapy [20], and the vinca alkaloid VCR is currently part of first-line chemotherapy protocols for the treatment of ES [3, 4]. However, microtubule-interfering agents are well-known for their side effects [16, 20] and VCR in particular frequently causes dose-limiting peripheral neuropathy [16, 49]. In contrast, eribulin is associated with a comparatively low incidence of severe peripheral neuropathy [16, 24], which has been linked to eribulin's different mode of microtubule binding and action as compared to other microtubule-targeting drugs [15, 16, 22].

Third, eribulin might overcome resistance to other microtubule-interfering drugs because of its distinct binding and mechanism of action towards microtubules [15, 23]. Indeed, it has already gained FDA approval for unresectable or metastatic liposarcoma after anthracycline failure as well as third-line therapy for metastatic breast cancer after failure of an anthracycline- and a taxan-containing regimen [23, 27].

Fourth, eribulin might suppress the development of metastasis, since it has recently been demonstrated to decrease metastasis of breast cancer cells in an experimental *in vivo* metastasis model which has been linked to the reversal of epithelial-mesenchymal transition [50]. This is of high relevance, since ES is often associated with early metastasis and metastatic disease correlates with poor survival [1–4, 51, 52].

Fifth, the eribulin concentrations that we used in the present study are presumably achievable in clinical practice, because a phase I trial demonstrated peak plasma concentrations above 2 nM after systemic application of eribulin [48].

Sixth, combined treatment of eribulin and PLK1 inhibitors might represent a promising therapeutic option for ES patients, because PLK1 has been shown to be overexpressed in different malignancies including ES [11]. The PPTP previously demonstrated that monotherapy of BI 6727 caused no objective *in vivo* responses in ES xenografts [53] highlighting the relevance of combination regimens. Currently, BI 6727 is being investigated in a phase I study for pediatric leukemias or advanced solid tumors without another known treatment option (www.clinicaltrials.gov).

Taken together, our findings indicate that eribulin alone or in combination with PLK1 inhibitors represents a promising strategy for the treatment of ES.

## MATERIALS AND METHODS

### Cell culture and chemicals

ES cell lines were kindly provided by C. Roessig (Muenster, Germany) or obtained from German Collection of Microorganisms and Cell Cultures (Braunschweig, Germany) or American Type Culture Collection (Manassas, VA, USA) and authenticated by STR profiles. Cells were maintained in DMEM GlutaMAX^™^-l or RPMI 1640 GlutaMAX^™^-l medium (Life Technologies, Inc., Darmstadt, Germany), supplemented with 10% fetal calf serum (FCS), 1% penicillin/streptomycin and 1 mM sodium pyruvate. Cell lines were regularly tested for mycoplasma contamination to guarantee that experiments were performed only with mycoplasma-free cells. For colony formation assay, cells were seeded on collagen-coated plates. Eribulin was obtained from Eisai Inc. (Frankfurt, Germany), zVAD.fmk from Bachem (Heidelberg, Germany) and bortezomib from Selleckchem (Munich, Germany). PLK1 inhibitor BI 6727 was kindly provided by Boehringer Ingelheim (Vienna, Austria) and TRAIL receptor-2 agonistic antibody ETR2 from Human Genome Sciences (Rockville, MD, USA). Chemicals were purchased from Sigma-Aldrich or Carl Roth (Karlsruhe, Germany) unless otherwise indicated.

### Determination of apoptosis and colony formation

Apoptosis was determined by flow cytometric analysis (FACSCanto II, BD Biosciences, Heidelberg, Germany) of DNA fragmentation of propidium iodide (PI)-stained nuclei as described previously [39, 54]. For colony formation assay, 100 cells (A4573) or 200 cells (SK-ES-1) per well were seeded in 6-well plates and treated for 15 hours (eribulin alone) or 18 hours (eribulin/BI 6727 co-treatment) with indicated drug concentrations. Subsequently, drug-containing medium along with detached, dead cells was removed and replaced by fresh medium. Cells were then cultured in drug-free medium for additional 9 days (A4573) or 12 days (SK-ES-1) before fixation and staining with crystal violet solution (0.5% crystal violet, 30% ethanol, 3% formaldehyde). Colonies were counted using ImageJ software (1.48v, National Institutes of Health, USA).

### Cell cycle analysis

Cells were stained with PI as described previously [55] and measured by flow cytometry. Cell cycle analysis was performed using FlowJo software (Tree Star Inc., Oregon, USA) following the manufacturer's instructions.

### Western blot analysis

Western blot analysis was performed as described previously [54] using the following antibodies: BCL-2, BCL-x_L_ (BD Biosciences, New Jersey, USA), murine BCL-2 (Invitrogen), caspase-3, caspase-9 (Cell Signaling, Beverly, MA, USA), BAX NT, pH3, α-tubulin (Millipore, Darmstadt, Germany), MCL-1, caspase-8 (Enzo Life Science, Lörrach, Germany), H3 (Abcam, Cambridge, UK), GAPDH (HyTest, Turku, Finland) and β-actin (Sigma-Aldrich). Goat anti-mouse IgG and goat anti-rabbit IgG conjugated to horseradish peroxidase (Santa Cruz Biotechnology, Heidelberg, Germany) as secondary antibodies and enhanced chemiluminescence (Amersham Bioscience, Freiburg, Germany) or infrared dye-labeled secondary antibodies and infrared imaging (Odyssey Imaging System, LI-COR Bioscience, Bad Homburg, Germany) were used for detection. Representative blots of at least two independent experiments are shown.

### Analysis of BCL-2 and BCL-x_L_ phosphorylation status

A4573 cells were treated for 15 hours with 2 nM eribulin, protein lysates were prepared and 50 μg protein were incubated with 50 U λ-phosphatase (400,000 U/mL in 50 mM Tris-HCl, 0.1 mM EGTA, 0.01% BRIJ 35 and 50% glycerol, pH 7.5, Santa Cruz), 1-fold λ-phosphatase buffer (50 mM HEPES, 0.1 mM EGTA, 5 mM dithiothreitol and 0.01% BRIJ 35, pH 7.5, Santa Cruz) and 2 mM MnCl_2_ (Santa Cruz) for 30 minutes at 30°C. Afterwards 6-fold SDS loading buffer was added, samples were denaturized for 5 minutes at 96°C, loaded on a SDS-PAGE gel and analyzed by Western blotting.

### Overexpression of murine BCL-2

Stable overexpression of murine BCL-2 was performed by using lentiviral vectors. Briefly, Phoenix cells were transfected with 20 μg of pMSCV plasmid (empty vector; BCL-2) using calcium phosphate transfection. Virus-containing supernatant was collected, sterile-filtered and used for spin transduction at 37°C in the presence of 8 μg/mL polybrene. For selection of transduced ES cells, 10 μg/mL blasticidin (Carl Roth) was used. Efficiency of transduction was confirmed by Western blotting.

### Determination of BAX activation

BAX activation was determined by immunoprecipitation using active conformation-specific antibodies. Briefly, cells were lysed in CHAPS lysis buffer (10 mmol/L HEPES, pH 7.4; 150 mmol/L NaCl; 1% CHAPS). 500 μg protein were incubated overnight at 4°C with 8 μg mouse anti-BAX antibody (clone 6A7; Sigma-Aldrich) and 10 μL panmouse IgG Dynabeads (Dako, Hamburg, Germany), washed with CHAPS lysis buffer and analyzed by Western blotting using rabbit anti-BAX NT antibody.

### Statistical analysis

Statistical significance was assessed by Student's *t*-Test (two-tailed distribution, two-sample, equal variance) using Microsoft Excel (Microsoft Deutschland GmbH); n.s. not significant; *p < 0.05; **p < 0.01; ***p < 0.001.

## SUPPLEMENTARY FIGURES



## Abbreviations

AML: acute myeloid leukemia
ES: Ewing sarcoma
EV: empty vector
FCS: fetal calf serum
FDA: Food and Drug Administration
NB: neuroblastoma
PC: positive control
PI: propidium iodide
PLK1: polo-like kinase 1
PPTP: Pediatric Preclinical Testing Program
RMS: rhabdomyosarcoma
TRAIL: Tumor-Necrosis-Factor-related apoptosis-inducing ligand
VCR: vincristine
zVAD.fmk: N-benzyloxycarbonyl-Val-Ala-Asp-fluoromethylketone
