# Supplementary material for: Eribulin alone or in combination with the PLK1 inhibitor BI 6727 triggers intrinsic apoptosis in Ewing sarcoma cell lines
Source: Oncotarget. 2017 Apr 18;8(32):52445–56. doi: 10.18632/oncotarget.17190 (PMC5581041; doi:10.18632/oncotarget.17190)
Supplement: Supplementary file 1 [file oncotarget-08-52445-s001.pdf]

Eribulin alone or in combination with the PLK1 inhibitor BI 6727 triggers intrinsic apoptosis in Ewing sarcoma cell lines

Supplementary Materials

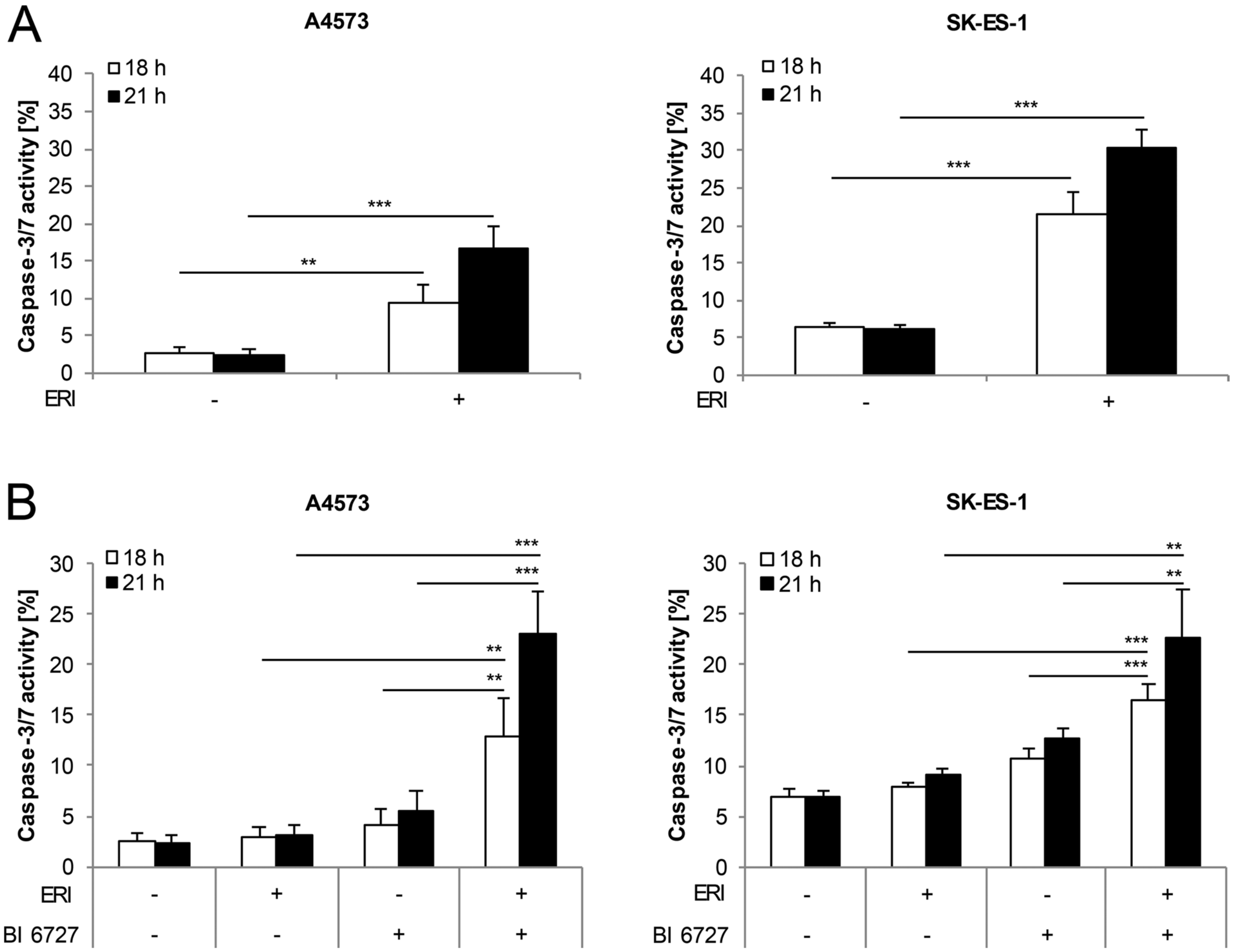

Supplementary Figure 1: Eribulin and eribulin/BI 6727 co-treatment trigger caspase-3/7 activity. (**A–B**) A4573 cells were treated with 2 nM eribulin (ERI) (A) or 0.4 nM eribulin and/or 15 nM BI 6727 (B), SK-ES-1 cells with 1 nM eribulin (A) or 0.15 nM eribulin and/or 15 nM BI 6727 (B) for indicated times. Caspase activity was determined by Cell Event Caspase-3/7 Green Detection Reagent and ImageXpress Micro XLS system following manufacturer's instructions. Data are shown as mean with SD of at least three independent experiments performed in triplicate; ** $p < 0.01$; *** $p < 0.001$.

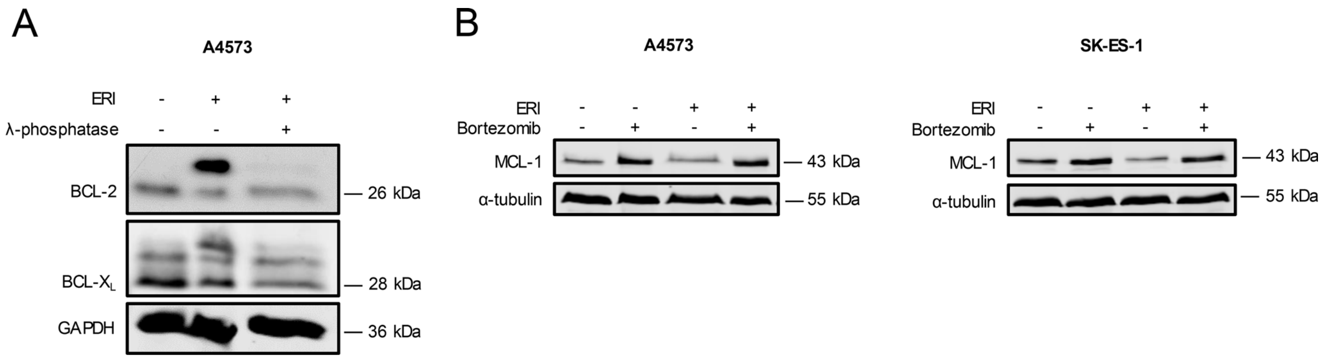

Supplementary Figure 2: Effect of λ-phosphatase on BCL-2 and BCL-x$_L$ phosphorylation and bortezomib on MCL-1 degradation. (**A**) A4573 cells were treated with 2 nM eribulin (ERI) for 15 hours, protein lysates were prepared and incubated or not with 50 U λ-phosphatase. Expression levels and phosphorylation status of BCL-2 and BCL-x$_L$ were assessed by Western blotting. GAPDH served as loading control. Representative blots of two independent experiments are shown. (**B**) A4573 and SK-ES-1 cells were pre-incubated for 1 hour with or without 10 nM bortezomib and subsequently treated for 15 hours with 2 nM eribulin (ERI) (A4573) or 1 nM eribulin (SK-ES-1) in the presence or absence of 10 nM bortezomib. Expression levels of MCL-1 were analyzed by Western blotting. Expression of α-tubulin served as loading control. Representative blots of three independent experiments are shown.

MATERIALS AND METHODS

Determination of caspase activity

Caspase activity was determined by Cell Event Caspase-3/7 Green Detection Reagent (Thermo Fisher Scientific, Darmstadt, Germany) and ImageXpress Micro XLS system.
